# Biomechanical behavior of fiber-reinforced and bulk-fill composites in extensive class II restorations: a three-dimensional finite element analysis

**DOI:** 10.2340/biid.v13.46067

**Published:** 2026-05-22

**Authors:** Luisfelipe Carlos Alarco-Jurado, Luis Felipe Alarco-La Rosa, Fredy Hugo Cruzado-Oliva

**Affiliations:** a Medical Sciences Unit, Doctoral Program in Stomatology, Graduate School, Universidad Nacional de Trujillo, Trujillo, Peru; b School of Stomatology, Faculty of Medicine, Universidad Privada Antenor Orrego, Trujillo, Peru; c School of Stomatology, Faculty of Stomatology, Universidad Nacional de Trujillo, Trujillo, Peru

**Keywords:** Finite element analysis, biomechanics, dental restoration, biomimetic restorative material, fiber-reinforced composite

## Abstract

**Introduction:**

Owing to the complexity of dental architecture and the ongoing development of restorative materials, the biomechanical behavior within restored dental structures is not yet fully understood.

**Objective:**

This study aimed to evaluate and compare the stress distribution in extensive Class II restorations reinforced with different composite systems using 3D-Finite Element Analysis (FEA).

**Materials and Methods:**

A sound human mandibular first molar was digitized to generate a 3D solid model. Five experimental groups with mesio-occluso-distal (MOD) restorations were simulated: G1, bulk-fill composite core; G2, short fiber–reinforced composite (SFRC) core; G3, ultra-high molecular weight polyethylene (UHMWPE) fiber–reinforced core; C−, conventional nanohybrid composite; and C+, intact sound tooth. All restored groups received an occlusal and proximal veneering layer of conventional nanohybrid resin. A vertical load of 600 N was applied, and von Mises and maximum principal stresses were calculated.

**Results:**

The UHMWPE group exhibited the most favorable stress distribution, demonstrating the lowest stress concentrations in the coronal tooth structure (353.5 MPa) and the adhesive interface (11.10 MPa). Conversely, the SFRC group presented the highest stress values in the remaining tooth structure (359.9 MPa), while the bulk-fill group recorded elevated stress peaks within the veneering material (21.29 MPa). Notably, the polyethylene core absorbed the highest internal stress (13.31 MPa) compared to the other experimental groups.

**Conclusions:**

The type of structural reinforcement significantly dictates the biomechanics of Class II restorations. A core reinforced with polyethylene fibers (Ribbond THM) demonstrates a superior capacity to manage stress in high-load areas compared to SFRC (EverX Posterior) and bulk-fill composite (Tetric N-Ceram bulk fill).

## Introduction

Today, the direct use of particle-filled or conventional composites (PFCs) is a common conservative approach for restoring missing dental structures [1]. Clinicians routinely use these materials in both anterior and posterior regions due to their excellent esthetics and favorable mechanical properties [2]. However, subsequent restorations present significant mechanical challenges during function, particularly in extensive preparations with proximal involvement. The loss of one marginal ridge can reduce tooth stiffness by 46%, and the loss of both ridges can reduce it by 63%. In other words, the greater the loss of tooth structure – whether from cavities, previous restorations, or fractures – the weaker the tooth and its restorations become [3].

A systematic review comparing the clinical success of direct restorations in posterior teeth between studies conducted from 1995 to 2005 and 2006 to 2016 reported survival rates of 89.4% and 86.9%, respectively. Failure modes in 1995–2005 included secondary caries (29.47%) and composite fracture (28.84%), compared with 2006–2016, where failures were attributed to secondary caries (25.68%) and composite fracture (39.07%) [4]. The likely explanation for the increase in fractures is the use of PFCs in extensive restorations [5].

More than 50 years after resin composites were first introduced into clinical practice, current research efforts continue to focus on optimizing the physicochemical properties of these materials through the incorporation of new monomers, fillers, initiators, adhesion promoters, and various artificial fibers as reinforcements, all aimed at improving the toughness of PFCs – a property that plays a major role in structural performance [6–8]. One of the earliest promising developments was the introduction of bulk-fill resin composites, which offer reduced polymerization shrinkage stress and improved fracture resistance. These materials can be applied in thicker increments and polymerized in a single step [9], reducing the risk of voids between layers compared with incremental techniques [10]. Additionally, restoring larger cavities in a single increment saves valuable clinical time [11].

Subsequently, short fiber-reinforced composites (SFRCs), which incorporate micro- or millimeter-scale glass fibers, emerged as a potential solution [12]. Researchers have stated that these materials represent the closest structural and mechanical analogue to dentin among composite modifications. Structurally, the random integration of short fibers within the composite matrix mimics the arrangement of collagen fibers in dentin. Mechanically, their flexural modulus, tensile strength, and fracture toughness are similar to those of dentin [1, 13, 14].

Over the past two decades, ultrahigh-molecular-weight polyethylene fibers woven in a leno pattern – also known as continuous or long fibers – have been introduced for various direct restorative techniques. Polyethylene fibers serve multiple purposes: first, to create a stress-absorbing layer that redirects potential cracks and fractures; and second, to internally splint the tooth and enhance fracture resistance. Application techniques involve placing the fiber along the cavity walls [15].

These materials require a conventional PFC coating layer on the occlusal and proximal surfaces in Class II restorations, as recommended by manufacturers. This coating layer acts as the missing enamel structure and is justified by its superior wear resistance and polishability compared with restorations lacking this layer [16].

Class II restorations performed by clinicians using different restorative materials can be evaluated through clinical trials, which provide a high level of scientific evidence regarding the quality and behavior of these materials. However, clinical studies require long follow-up periods, are difficult to perform, and are costly. Therefore, laboratory tests serve as valuable evaluation tools, allowing comparison of materials under controlled conditions. Nevertheless, traditional laboratory tests are destructive and do not allow detection of initial crack formation during failure processes [17].

Finite element analysis (FEA) studies are increasingly accepted as a valuable method for understanding biomechanical properties that cannot be examined using standard in vitro experimental models. In dentistry, FEA has been used mainly to achieve a more detailed understanding of the biomechanical performance of restorative systems, as well as the distribution of stress and strain in vulnerable areas prone to fracture, including the tooth-restoration interface [18].

To date, there is a gap in the literature regarding a direct comparison of the biomechanical behavior between bulk-fill composite cores, short glass fibers, and polyethylene fibers under standardized load conditions in Class II cavities. The uncertainty surrounding how these reinforcement materials interact with the remaining tooth structure and the adhesive layer justifies the need for a detailed computational study. Therefore, the objective of the present investigation was to evaluate and compare, via 3D-FEA, the stress distribution in the remaining tooth structure, the adhesive layer, and the restorative complex of Class II cavities treated with three reinforcement systems: a bulk-fill composite (Tetric N-Ceram), a short fiber-reinforced composite (EverX Posterior), and ethylene polyfibers (Ribbond THM), all protected by a nanohybrid composite veneering layer. The null hypothesis was that there would be no significant differences in Von Mises stress patterns or maximum principal stress values across the remaining tooth structure, the adhesive interface, or the restorative complex, regardless of the reinforcement system used.

## Materials and methods

### Study design

The present study was an experimental, cross-sectional, comparative, in vitro investigation. It was conducted using FEA, in which the study variable was the MOD restoration performed with bulk-fill composite (Tetric N-Ceram), short glass fiber–reinforced composite (EverX Posterior), and polyethylene fiber–reinforced resin (Ribbond THM). In addition, two control groups were created: an intact, healthy tooth and a tooth restored with a MOD preparation using PFC (Grandio). The materials used in this study are detailed in Table 1.

**Table 1:** Technical specifications and chemical composition of the materials used in the study.

Material	Material category	Matrix composition	Filler type	Filler content (wt% / vol%)	Manufacturer
Tetric N-Ceram bulk fill	Bulk-fill composite resin	Bis-GMA, UDMA, Bis-EMA.	Barium glass, ytterbium trifluoride, mixed oxides	75–77% / 53–55%	Ivoclar Vivadent AG, Schaan, Liechtenstein
EverX Posterior	Short Fiber Reinforced Composite (SFRC)	Bis-GMA, TEGDMA, PMMA	E-glass fibers (short) and barium particles	76% / 57%	GC Corporation, Tokyo, Japan
Ribbond THM	Structural reinforcing fiber	Ultra-high molecular weight polyethylene (UHMWPE)	Plasma-treated woven fiber (dense weft)	N/A (pure fiber)	Ribbond Inc., Seattle, WA, USA
Grandio	Nanohybrid composite	Bis-GMA, TEGDMA.	Silicon dioxide, aluminum, and barium borosilicate glass	87% / 71.4%	VOCO GmbH, Cuxhaven, Germany
Tetric Flow	Flowable resin composite	Dimethacrylates (Bis-GMA, UDMA, TEGDMA)	Barium glass, ytterbium trifluoride, highly dispersed silica, and mixed oxide	63 % / 39 %	Ivoclar Vivadent AG, Schaan, Liechtenstein
Clearfil SE	Two-step self-etch adhesive	10-MDP, Bis-GMA, HEMA, hydrophilic dimethacrylates	Colloidal silica (nanofillers)	~10% / ~5%	Kuraray Noritake Dental Inc., Tokyo, Japan

### Geometric modeling and solid preparation

A recently extracted human mandibular first molar, removed for periodontal reasons, was selected for this study. To ensure anatomical representativeness, the morphometric integrity of the specimen was verified according to standard dental dimensions: coronal length (8 mm), mesiodistal width (11 mm), buccolingual width (10.5 mm), and root length (13 mm).

The digital geometry was acquired using a high-resolution multifunctional 3D dental scanner (UP560; Shenzhen UP3D Tech Co., Shenzhen, China), generating a point cloud exported in STL (Standard Tessellation Language) format. The initial STL mesh was processed using Meshmixer (Autodesk Inc., San Rafael, CA, USA) for surface refinement, noise reduction, and smoothing to ensure geometric continuity and eliminate topological artifacts.

The refined mesh was subsequently imported into SOLIDWORKS (Dassault Systems, Velizy-Villacoublay, France), where it was converted into a NURBS-based (Non-Uniform Rational B-Splines) solid model. This conversion facilitated precise volumetric operations, such as the simulation of the MOD cavity preparation and the accurate identification of anatomical reference planes within a standardized three-dimensional coordinate system. This workflow ensured that the volumetric transition between the different restorative materials and the dental substrates was mathematically continuous for the meshing phase.

### Cavity design and restorative simulation

**Figure 1:**
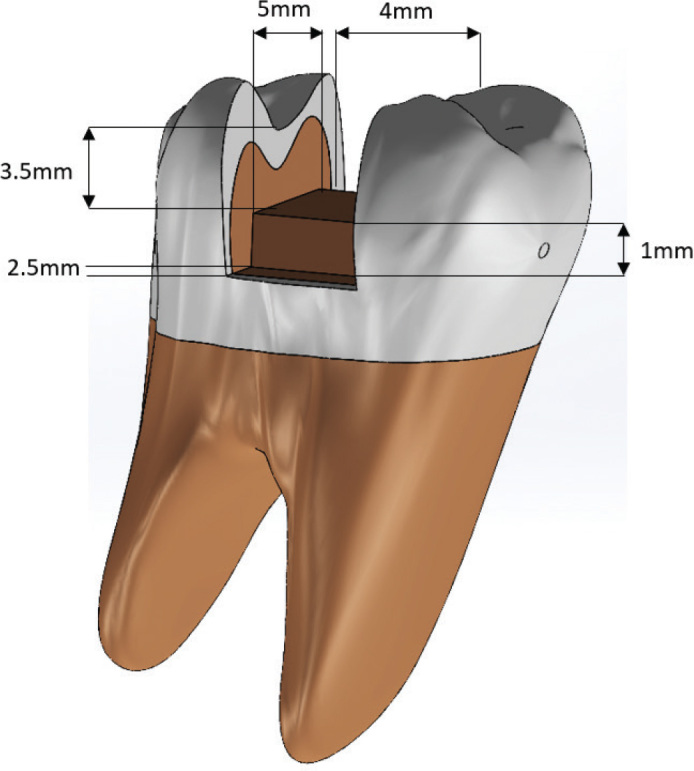
Three-dimensional model and geometric specifications of the Class II (MOD) cavity preparation.

A standardized MOD cavity preparation was digitally engineered on the solid model using Boolean subtraction operations. The preparation adhered to strict biometric parameters: a pulpal floor depth of 3.5 mm from the central groove, a buccolingual width of 4 mm, and a cervical step positioned 2.5 mm from the proximal surfaces with an axial thickness of 1 mm (Figure 1).

**Figure 2:**
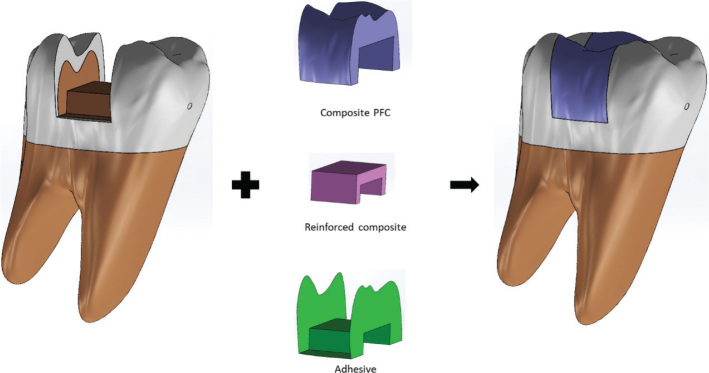
Structural architecture of the restored model and layer-by-layer component integration.

To simulate realistic clinical conditions, an adhesive layer with a 50 µm thickness was modeled at the tooth-restoration interface [19]. The restorative complexes were designed using a bilayer approach: The Core: A base consisting of the experimental material (bulk-fill composite, SFRC, or polyethylene fiber reinforcement). The Veneer: An enamel-replacement layer comprising a 2 mm occlusal and 1.5 mm proximal thickness of conventional nanohybrid composite (Grandio), in accordance with manufacturer recommendations (Figure 2).

### Discretization phase and simulation environment

The volumetric assemblies were exported in STEP format and imported into the FEA suite ANSYS (ANSYS Inc., Canonsburg, PA, USA). Each restorative component was defined as an independent volumetric entity, allowing for the assignment of specific mechanical properties. To simulate an integral adhesive bond, ‘Bonded’ contact conditions (perfect adhesion) were defined at all interfaces between the dental substrates and the restorative materials.

The discretization (meshing) process was executed using high-order 3D quadratic tetrahedral elements (10-node elements), which provided superior accuracy in representing complex dental geometries compared to linear elements. A mesh convergence analysis was performed for each model to ensure that the stress results were independent of the mesh density. The final number of nodes and elements for each experimental model, validated after achieving convergence, is detailed in Table 2.

**Table 2:** Mesh statistics: Number of nodes and elements for the simulated molar models.

Simulated Models	Number of nodes	Number of elements
Intact tooth	312,249	182,080
Restoration with Grandio	380,961	217,414
Restoration with Tetric N-Ceram bulk fill	428,543	237,790
Restoration with EverXPosterior	427,316	230,418
Restoration with Ribbond THM	428,543	237,790

### Experimental groups and configuration

**Figure 3:**
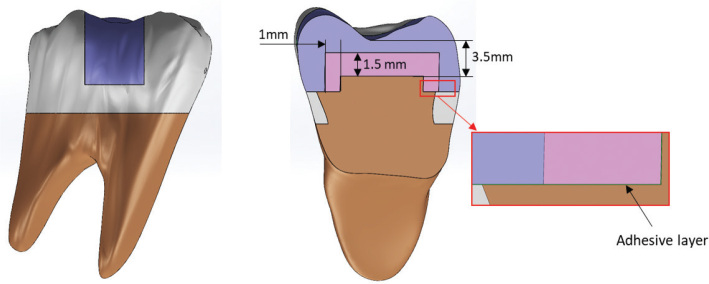
Cross-sectional configuration and internal dimensions of the multilayered restoration design.

Five distinct computational models were developed to analyze biomechanical behavior within the standardized MOD cavity framework. The groups were categorized as follows (Figure 3):

### Experimental groups (reinforced cores)

G1 (bulk-fill): Core build-up utilizing a bulk-fill composite (Tetric N-Ceram bulk fill), finished with a peripheral veneering layer of conventional nanohybrid resin (Grandio).

G2 (SFRC): Core build-up utilizing an SFRC (EverX Posterior), finished with a peripheral veneering layer of conventional nanohybrid resin (Grandio).

G3 (UHMWPE): Core build-up reinforced with ultra-high molecular weight polyethylene fibers (Ribbond THM) and finished with a peripheral veneering layer of nanohybrid resin (Grandio). This group simulated the ‘Wallpapering’ technique through a 1.5-mm homogenized biomimetic layer at the cavity base. To ensure clinical realism, this layer was modeled as UHMWPE fibers intimately embedded within a flowable resin matrix (Tetric Flow; Ivoclar Vivadent AG, Schaan, Liechtenstein).

### Control groups

Control negative (C−): An MOD restoration fabricated entirely from conventional nanohybrid resin (Grandio), representing the standard restorative protocol without structural reinforcement.

Control positive (C+): An intact, sound molar model used to establish the baseline for natural structural stiffness and physiological stress distribution.

### Material properties and model assumptions

For the computational analysis, all dental tissues and restorative materials were assumed to be isotropic, homogeneous, and linearly elastic [20, 21]. This simplification is an established standard in dental FEA research under infinitesimal strain conditions, ensuring model stability and comparability.

The assigned mechanical properties, specifically Young’s Modulus (*E*) and Poisson’s ratio (*v*), are synthesized in Table 3.

**Table 3:** Mechanical properties and elastic constants of the materials used in the finite element analysis.

Component	Modulus of elasticity (GPa)	Poisson’s ratio
Enamel	84.1	0.3 [22]
Dentine	18.6	0.31 [22]
Pulp	2.0	0.45 [22]
Food bolus	3.4	0.1 [23]
Clearfil SE adhesive layer	0.39	0.32 [24]
Universal nanohybrid composite (Grandio)	20.4	0.33 [21]
Bulk-fill reinforced composite (Tetric N-Ceram bulk fill)	10	0.24 [25]
Short glass fiber reinforced composite (EverXPosterior)	11.4	0.24 [22]
Composite reinforced with polyethylene fibers (Ribbond THM + bonding agent + Tetric Flow)	23.6	0.32 [21]

### Boundary conditions and loading protocol

The model’s support was established by applying a fixed constraint to the root bases in all three spatial axes (x, y, z), simulating physiological alveolar anchorage. To faithfully replicate mandibular kinematics, a contact body was designed to simulate a food bolus. This body was strategically positioned in contact with the middle third of the occlusal surface and the reconstructed marginal ridges. A static load of 600 N was applied vertically, parallel to the longitudinal axis of the tooth. This magnitude represents the upper limit of maximum masticatory forces recorded in humans [22, 23]. The load was transmitted through the food bolus interface to the occlusal surface, simulating the phase of maximum intercuspation during the masticatory cycle. This approach ensures a more realistic stress distribution compared to localized point loading.

## Results

**Figure 4:**
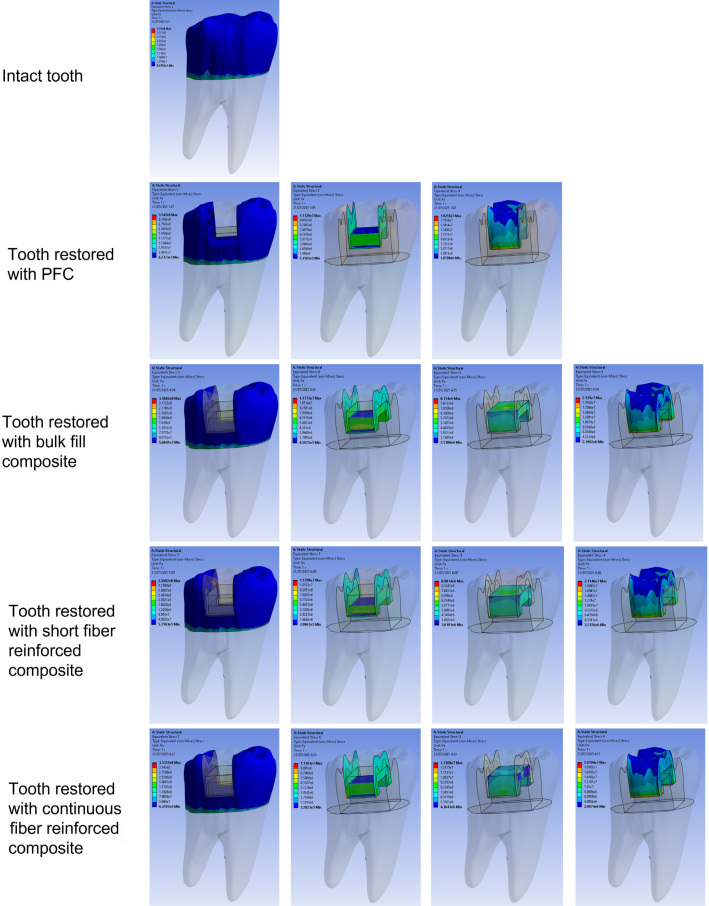
Von Mises stress distribution maps across the groups under occlusal loading.

The Von Mises stress contour maps revealed distinct load distribution patterns across the experimental groups (Figure 4). In the negative control group (C−), critical stress concentrations were localized at the occlusal margins of the restoration, with a relatively uniform distribution throughout the adhesive layer, albeit with stress peaks at the dihedral angles. In restorations utilizing a bulk-fill composite core, stresses shifted toward the proximal walls and the cervical step, indicating a higher load transfer toward the base of the cavity. Conversely, the SFRC group (EverX Posterior) exhibited slight optimization in distribution homogeneity compared to the bulk-fill group, effectively reducing stress gradients at the proximal interface.

The most favorable biomechanical behavior was observed in the UHMWPE group (Ribbond THM). This model presented the most balanced stress distribution across all system components, minimizing areas of hyper-concentration within the remaining tooth structure and dissipating loads more efficiently through the reinforced core.

The sound tooth (positive control) displayed a baseline stress of 351.0 MPa. Among the restored groups, the polyethylene fiber reinforcement generated the lowest stress increase in the dental tissue (353.5 MPa), closely approximating the natural tooth’s behavior. In contrast, short glass fibers induced the highest stress levels in the remaining structure (359.9 MPa).

Regarding the adhesive layer, the highest stress levels were recorded in the SFRC (11.80 MPa) and bulk-fill (11.78 MPa) groups. The inclusion of polyethylene fibers reduced this stress to 11.10 MPa, suggesting a decreased risk of adhesive interface failure.

Within the restorative core, the polyethylene fiber core sustained the highest internal stress (13.31 MPa) compared to the glass fibers (8.99 MPa) and the bulk-fill resin (8.31 MPa). In the veneering composite, the conventional nanohybrid resin reached its peak stress in the bulk-fill group (21.29 MPa), while remaining more stable in the fiber-reinforced models. Comprehensive data for the maximum stress values are detailed in Table 4.

**Table 4:** Maximum stress values (MPa) recorded in the tooth structure and restorative materials for each study group.

Experimental Groups	Coronary remnant	Adhesive	Coating composite	Reinforced composite
Intact tooth	351.0	------	------	------
Tooth restored with Grandio	354.2	11.13	19.26	------
Tooth restored with Tetric N-Ceram bulk fill	356.8	11.78	21.29	8.31
Tooth restored with EverXPosterior	359.9	11.80	21.15	9.99
Tooth restored with Ribbond THM	353.5	11.10	20.10	13.31

## Discussion

The management of structurally compromised teeth remains a subject of ongoing debate in restorative dentistry. Procedures involving post-endodontic reconstruction, the replacement of large restorations, and the management of extensive carious lesions are staples of daily clinical practice, necessitating constant innovation in reinforcement strategies [2]. This study analyzed the stress distribution of extensive Class II restorations fabricated with various reinforced composite systems. Based on our findings, the null hypothesis was rejected, as the analysis demonstrated that the type of structural reinforcement significantly alters the biomechanical behavior of the tooth-restoration complex.

In the sound tooth model (positive control), stress distribution was homogeneous and primarily concentrated within the peripheral enamel, with no areas of hyper-concentration in the deep dentin. This finding aligns with previous reports emphasizing the natural tooth’s efficiency in dissipating occlusal loads through its hierarchical structure [22]. However, the biomechanics change drastically in extensive Class II preparations.

In the model restored with a conventional nanohybrid composite (Grandio), stress patterns were uniform within the adhesive layer, but elevated stress peaks were observed at the cavity margins. This phenomenon is attributed to the material’s high rigidity; lacking an internal energy-absorption mechanism, it transmits the load directly toward the line angles and the adhesive interface, thereby increasing the risk of marginal fracture and bond degradation [20]. Indeed, it is well established that polymerization and functional stress tend to increase with composite stiffness. Grandio, being a conventional nanohybrid resin with a relatively high elastic modulus, exhibits reduced load-damping capacity, which correlates with the higher marginal tension observed in this study [22, 26].

In the experimental groups, the stress distributions were shown as follows:

In the remaining tooth structure, the UHMWPE group (Ribbond THM) exhibited the lowest stress transfer, whereas the SFRC group (EverX Posterior) recorded the highest values. These findings suggest that continuous fiber reinforcement acts as a structural unit that absorbs a portion of the load or distributes it more uniformly, drastically reducing stress on the underlying dentin [27]. According to the ‘Wallpapering’ protocol by Deliperi et al., fibers bonded to the dentinal walls modify interfacial stresses by acting as ‘stress bridges’ that extend load trajectories, favoring repairable fracture patterns above the gingival line [28]. This damping effect explains the stress reduction in the UHMWPE group.

Conversely, the SFRC group exhibited higher stress peaks, indicating that this restoration was overall stiffer and transmitted more load to the tooth through friction mechanisms between the short fibers and the matrix [29].

Nevertheless, the SFRC maintained a superior protective profile compared to the bulk-fill composite, corroborating reports on the efficacy of short fibers in extensive cavities [16].

In the adhesive layer, all restored models presented similar stress levels. This indicates that the thickness and properties of the adhesive remained constant, fulfilling its role as a ‘stress-grading zone’. Biomechanical studies suggest that a more flexible adhesive can absorb polymerization shrinkage [26], and increasing its thickness can reduce stress accumulation [30]. In our study, adhesive stress was moderate and uniform, aligning with results by Duarte et al. [31]. The minimal variation between groups suggests that the observed differences are focused on the restorative materials rather than the adhesive interface, consistent with Mohammadi et al. [19]. However, current evidence suggests that employing cohesive zone modeling (CZM) to include the adhesive interface provides a superior understanding of how cavity geometry dictates fracture strength, as well as a more detailed analysis of the debonding process, particularly in extensive MOD cavities where the interface is subjected to complex stress states [32, 33].

Regarding the veneering layer, the bulk-fill group showed the highest stress peaks, followed closely by the SFRC group. This is likely due to the discrepancy between the elastic modulus and polymerization shrinkage of the core versus the veneering composite, generating shear stress at the interface. Previous studies have reported that bulk-fill resins, although designed for lower shrinkage, can exhibit high internal stresses depending on the loading mode or curing depth [34]. Indeed, Fidancioğlu et al. [20] and Tuncdemir et al. [35] found that bulk-fill restorations produced higher enamel stress peaks than amalgam, demonstrating that the lower elastic modulus of some bulk-fills results in less load absorption and higher stress transmission to the veneering composite. The increased tension in the proximal walls of the bulk-fill group reflects this trend. The UHMWPE group presented the lowest stress in this layer, indicating that the continuous fibers assumed a larger portion of the load due to their high linear tenacity and the interlocking network of the Ribbond THM [36].

The reinforced cores exhibited distinct behaviors. The SFRC group showed intermediate stress levels – lower than the bulk-fill but higher than the UHMWPE. Short fiber-reinforced resins are designed to distribute stress via dispersed microfibers, acting as ‘crack stoppers’; however, being discontinuous, they do not function as full structural beams [37]. In contrast, the UHMWPE group presented the maximum stress values within the reinforced region itself, indicating that the continuous fibers ‘trap’ and support the exerted loads. This high internal stress is consistent with its function as a load-bearing element, where its interwoven nodes allow for efficient load transfer [20].

Consequently, Ribbond THM behaved as the primary resistance component, where the increase in core stress coincided with a decrease in stress in other areas (adhesive, tooth, and veneer). These results support a ‘fail-safe mechanism’, where long-fiber reinforcements tend to fracture within the material first, preserving the underlying structural integrity. This behavior illustrates the stress-shielding mechanism [28].

Despite these findings, recent systematic reviews have found no significant differences in fracture resistance between SFRCs and bulk-fill composites [38]. Similarly, an ex vivo study observed that SFRCs exhibited higher fracture resistance than both polyethylene-reinforced and bulk-fill resins [9]. Furthermore, 24-month clinical trials observed no significant differences between bulk-fill and polyethylene fiber-reinforced restorations in endodontically treated teeth [39].

This suggests that the clinical performance of reinforced materials depends on a multifactorial interplay involving the C-factor, cavity geometry, remaining tooth structure, loading mode/direction, composite volume, and reinforcement architecture (continuity, orientation, and placement). The stiffness gradient of the restorative system and the type of stress analysis employed also play pivotal roles in the observed outcomes.

### Clinical implications

The findings of this study have direct relevance for the selection of restorative materials in extensive Class II posterior cavities. This research provides clinicians with a stress-distribution-based guide for MOD restorations. The reduction of internal stress concentrations may translate into a lower probability of microcrack initiation, as well as a reduced risk of structural failure of both the tooth and the restoration, particularly under high functional loads.

Clinically, the use of fiber-reinforced materials can enhance biomechanical durability. Specifically, polyethylene fibers offer a more uniform stress redistribution, potentially leading to a lower risk of catastrophic fracture and marginal failure. While SFRCs also offer benefits – such as reduced fracture susceptibility and lower stress compared to conventional composites – continuous fiber reinforcement may be preferable in cases involving extreme loss of tooth structure. In contrast, bulk-fill composite restorations exhibited higher stress concentrations, suggesting that caution should be exercised when using these materials in very large cavity preparations. Overall, the integration of advanced materials (both continuous and short fiber-reinforced composites) represents a viable strategy for restoring extensively cavitated teeth while preserving the structural integrity of the remaining dental tissue.

## Limitations

Despite providing critical insights into the biomechanical behavior of various reinforcement systems, certain limitations must be acknowledged to contextualize the findings. As with all FEA studies, this model assumed linear-elastic behavior and did not simulate the real-time kinematics of polymerization shrinkage or the material viscoelasticity over time. Furthermore, all restorative materials and dental substrates were assumed to be isotropic and perfectly bonded. This represents a significant simplification of clinical reality, where fiber misalignment and potential microleakage are prevalent. Specifically, the assumption of perfect adhesion remains a limitation; the inclusion of advanced formulations, such as CZM, would provide a more realistic simulation of the adhesive joint interface and a more refined understanding of the biomechanical interaction between reinforcement systems and the dental substrate.

Regarding the loading conditions, the application of a purely vertical load represents a simplified worst-case scenario for maximum intercuspation. However, it does not fully capture the complexity of dynamic mandibular movements, including the lateral components and bending stresses typical of masticatory cycles. Similarly, factors such as cyclic fatigue, intraoral temperature fluctuations, and hydrolytic degradation – which significantly influence clinical longevity – were not considered in this static simulation.

Finally, the scope of this study was restricted to a standardized MOD cavity preparation. While this represents a high-risk clinical scenario for stress concentration, future research should explore diverse cavity geometries (e.g. Class I, Class V, or endodontic access) to assess the materials’ versatility under different structural challenges. Additionally, although the selected materials are representative of current clinical standards, investigating a broader range of adhesive systems and composite formulations would provide a more comprehensive understanding of the synergistic effects within the restorative complex. Therefore, while these results provide clear comparative trends, they should be interpreted qualitatively. Future research should complement these findings with experimental fracture resistance studies or dynamic, multi-vector loading simulations to validate these stress patterns under more realistic conditions.

## Conclusion

Within the limitations of this 3D-FEA, it is concluded that the incorporation of structural reinforcement in Class II (MOD) restorations significantly modifies the stress distribution within the tooth–restoration complex. Polyethylene fiber-reinforced composite (UHMWPE) exhibited the most favorable biomechanical behavior, demonstrating the lowest stress levels, followed by SFRC and bulk-fill composites.

## Data Availability

The data supporting the findings of this study are available from the corresponding author, FC-O, upon reasonable request.
